# Disfluencies as a Window into Pragmatic Skills in Russian-Hebrew Bilingual Autistic and Non-Autistic Children

**DOI:** 10.1007/s10803-024-06533-w

**Published:** 2024-09-19

**Authors:** Marianna Beradze, Natalia Meir

**Affiliations:** 1 The Department of English Literature and Linguistics, Bar-Ilan University, Ramat Gan, 5290002 Israel; 2 The Department of English Literature and Linguistics and the Gonda Brain Research Center, Bar-Ilan University, Ramat Gan, 5290002 Israel

**Keywords:** Autism, Disfluencies, Filled pauses, Bilingualism, Language-specific, Language-universal

## Abstract

There is little research on the production of speech disfluencies such as silent pauses, repetitions, self-corrections, and filled pauses (e.g., *eh*, *em*) in monolingual autistic children, and there is no data on this crucial part of speech production in bilingual autistic children. This study aims to address this gap by examining disfluency production in bilingual autistic and non-autistic children across two linguistically distinct languages, HL-Russian (the home language) and SL-Hebrew (the societal language). Fifty-one bilingual Russian-Hebrew-speaking autistic and non-autistic children aged 5–9 (autistic: *n* = 21; non-autistic: *n* = 30), matched for age and non-verbal intelligence, participated in picture-based story-generation tasks (LITMUS MAIN, Gagarina et al., ZAS Papers in Linguistics, 63:1–36, 2019). Audio recordings of narrative samples were transcribed, coded, and scored for eleven disfluency types using CLAN tools. The non-autistic group produced higher overall disfluency rate than the autistic group. The autistic group exhibited fewer filled and silent pauses than the non-autistic group in HL-Russian. Furthermore, non-autistic children manifested varied distribution of disfluency types across languages, while autistic children displayed more consistent patterns across languages. In summary, we replicated findings from previous research on monolinguals only partly, as no between-group difference in filled pauses was found in SL-Hebrew. Additionally, bilingual autistic children exhibited language-universal patterns of disfluency production, whereas their non-autistic peers displayed language-specific patterns.

**Supplementary Information:**

The online version contains supplementary material available at 10.1007/s10803-024-06533-w.

## Introduction

With a growing number of bilingual children alongside the increasing prevalence of autism spectrum disorders (ASD) (Prévost & Tuller, 2022), it becomes essential to integrate research on autism and bilingualism. Notably, studies on disfluency production by autistic children lack this integration, despite the limited scope of research available on disfluency production in monolingual autistic children.

The primary aim of this study was to address this gap by examining patterns of disfluency production in bilingual Russian-Hebrew-speaking autistic and non-autistic children aged 5–9, across their two linguistically distinct languages: HL-Russian (the home language) and SL-Hebrew (the societal language). This investigation broadened the scope of analyzed disfluencies compared to previous research to encompass eleven types, including filled pauses (*eh* and *em*), silent pauses, polysyllabic whole-word repetitions, phrase repetitions, whole-word self-corrections, phrase self-corrections, phonological fragments, part-word repetitions, monosyllabic whole-word repetitions, prolongations, and broken words, using the *Computerized Language Analysis* (CLAN) program (MacWhinney, 2000).

The second aim of this study was to examine whether there are between-group differences in the disfluency production of bilingual children and, specifically, whether autistic children exhibit fewer filled pauses than their non-autistic peers replicating previous findings that observed reduced occurrences of filled pauses in monolingual autistic individuals compared to non-autistic counterparts (Clin & Kissine, 2023; Gorman et al., 2016; Hallin et al., 2016; Heeman et al., 2010; Irvine et al., 2016; Jones et al., 2022; Lake et al., 2011; Lawley et al., 2022; Lunsford et al., 2010; MacFarlane et al., 2017; McGregor & Hadden, 2020; Morett et al., 2016; Parish-Morris et al., 2017; Salem et al., 2021).

Filled pauses serve various pragmatic functions in speech, primarily aiding in planning, as well as in maintaining conversational turns and emphasizing points and their production is notably influenced by the register and context (Kosmala, 2022). Recent insights from studies on disfluency indicate that filled pauses function as pragmatic markers, serving both textual and interpersonal purposes, and their frequency and functions are influenced by contextual factors in language production (Tonetti Tübben & Landert, 2022). The Special Issue by Beeching et al. (2022) consolidates research on discourse-pragmatic markers and filled pauses, suggesting that examining them together is highly relevant and timely. Therefore, considering that the production of discourse markers could pose difficulties for those “who struggle with neurotypical conventions about conversation reciprocity and other aspects of pragmatics, such as individuals on the autism spectrum (APA, 2013)” (Jones et al., 2022, p3), it is  conceivable that autistic individuals could potentially experience difficulties with producing filled pauses.

This research delves into disfluency production, proposing that examining the pragmatic functions of disfluencies in discourse may shed light on distinct disfluency patterns in both autistic and non-autistic populations. We will analyze our results following the perspective presented by Clin and Kissine (2023), which extends the study by Lake et al. (2011), suggesting that autistic individuals might sound different because they exhibit either fewer listener-oriented disfluencies (thus, being less helpful to the listener) or an increase in speaker-oriented disfluencies (potentially making the speech more complicated to follow). However, we will also explore alternative explanations, such as Lawley et al.'s (2022) suggestion that reduced filled pauses in autistic children may stem from challenges in speech production.

Third, this study aimed to elucidate the association between the disfluencies produced by both groups in both languages and various factors including language abilities, cognitive abilities, and mentalizing skills (such as Theory of Mind (ToM)) and the relationship between disfluencies and Autism Diagnostic Observation Schedule scores (ADOS; Lord et al., 2012) within the autistic group. Although there is partial overlap between pragmatics and ToM, neither can be regarded simply as a sub-component of the other. Several studies have shown a notable connection between ToM and pragmatic skills (Baixauli-Fortea et al., 2015; Cardillo et al., 2021; Kuijper et al., 2017; Losh et al., 2012). On the other hand, some studies conclude that pragmatics is a distinct construct from ToM (for more discussion see Bosco et al., 2018).

### Language-Universal and Language-Specific Aspects of Disfluency

The concept of speech disfluencies has been defined in various ways over time. In the 1950s, they were described as ‘extralinguistic events’ that disrupt the natural flow of speech, as pointed out by Wingate (1987). However, since the 1980s, various studies have begun to regard certain types of disfluencies as powerful communicative tools (Betz et al., 2023). In 1995, speech disfluencies were defined as “phenomena that interrupt speech flow but do not add propositional content to an utterance” (Fox Tree, 1995, p.1). Later, Fox Tree (2006) further refined the definition, stating that disfluencies are any of a group of phenomena that cause a break in the smooth flow of spoken talk. More recently, Clark (2013, p.1) noted that “disfluency is any feature of an utterance that deviates from the ideal delivery of that utterance.”

Fox Tree (1995) estimated the occurrence of disfluencies across different studies, excluding silent pauses, to be around 6%. However, it is possible to observe language-specific characteristics in the distribution of overall disfluency rates and types. For instance, the average number of disfluencies per 100 words produced by children in conversational tasks, was 10.6 for French-speaking children (mean age 50 months), 4.3 for English-speaking children (mean age 50.47 months), and 5.33 for Finnish-speaking children (mean age 48 months) (details in Jansson-Verkasalo et al., 2021). There was also variation in disfluency types across languages, such as filled pauses among 4- to 5-year-olds. The mean and standard deviation values were as follows: French, 3.68 (2.18); English, 2.04 (1.69); German, 0.74 (0.58); Spanish, 0.89 (0.55); and Finnish, 1.50 (1.62) (details in Jansson-Verkasalo et al., 2021; Leclercq et al., 2018).

Filled pauses are a nearly universal feature of language (Irvine et al., 2016). In a study by Clark and Fox Tree (2002) comparing eight languages, it was observed that each language has two or more filled pauses consisting of one or two syllables, which tend to be brief but can also be prolonged. Nevertheless, the phonetic realizations of filled pauses seem to be language-specific (see Clark and Fox Tree (2002) for a list of filled pauses across different languages). The prevalent types of filled pauses also appear to be language-specific. For instance, in American English, filled pauses like *uh* and *um* are commonly used, while in languages such as Swedish, Norwegian, Spanish, French, and Hebrew, filled pauses are most frequently realized as *eh* (Irvine et al., 2016; Schettino et al., 2022). Verkhodanova et al. (2018) found that among hesitations (filled pauses and prolongations) in Russian, *eh* is the most frequently produced filled pause, while the /i/ is the most frequently lengthened sound in Russian.

The frequent production of prolongations over the production of filled pauses in specific languages is an additional example of the language-specific production of disfluencies. Silber-Varod's (2013) examination of disfluencies within the Israeli Hebrew spontaneous speech corpus revealed that excessively prolonged word-final syllables constituted the most frequent disfluency type, followed by filled pauses, most of which were occurrences of the *eh.* Schettino et al. (2022) also revealed a common tendency for producing final-word prolongations over filled pauses in two closely related languages, Spanish and Italian, albeit with variations in how these phenomena are employed and phonetically realized between the two languages.

### The Production of Disfluencies in Monolingual Autistic Children

While the literature generally agrees that nearly all autistic individuals display distinctive pragmatic abilities compared to non-autistic individuals, it is essential to clarify that these differences do not encompass all aspects of pragmatics, nor are they consistent across all autistic individuals (Schaeffer et al., 2023). Considering the pragmatic challenges encountered by autistic individuals, the scope of research in the realm of disfluency has broadened to encompass a comparative analysis between autistic individuals and non-autistic individuals to assess whether both groups exhibit similar patterns of disfluency production. Among the pioneering studies in disfluency production by autistic individuals were those conducted by Heeman et al. (2010) and Lunsford et al. (2010), who expanded upon Clark and Fox Tree's (2002) suggestion that filled pauses may serve pragmatic functions, such as communicating to the listener that the speaker is searching for a word, constructing what to say next, or wishing to keep or cede the floor (Clark & Fox Tree, 2002). Heeman et al. (2010) and Lunsford et al. (2010) hypothesized that autistic children would produce  filled pauses differently from their non-autistic peers and confirmed this hypothesis by revealing a lower frequency of *um* in autistic children across a range of tasks. In their analysis, Lunsford et al. (2010) elucidated the differentiation between *um* and *uh*, attributing them to distinct cognitive processes: *um* arises from an externally focused process, where the speakers employ it to aid the listeners, while *uh* stems from an internally directed process, where the speakers use it to assist themselves. Subsequently, Lake et al. (2011) categorized various types of disfluencies as either listener- or speaker-oriented based on similar criteria suggested by Lunsford et al. (2010) for differentiation between *um* and *uh*. Observing that autistic adults exhibited fewer filled pauses and self-corrections and more silent pauses and repetitions, Lake et al. (2011) concluded that filled pauses and self-corrections are listener-oriented disfluencies (produced to aid the listener) while silent pauses and repetitions and speaker-oriented disfluencies (produced to aid the speaker) (see Table A, Online Resource 1).

*Filled Pauses*: In terms of filled pauses, most of the studies on monolingual autistic children and their non-autistic peers confirmed the results of Lake et al. (2011) demonstrating that autistic children and adolescents (hereafter children) consistently produce fewer listener-oriented filled pauses than their non-autistic peers (Gorman et al., 2016; Hallin et al., 2016; Irvine et al., 2016; Lawley et al., 2022; MacFarlane et al., 2017; McGregor & Hadden, 2020; Morett et al., 2016; Parish-Morris et al., 2017; Salem et al., 2021). Others found no difference in filled pauses (for Croatian-speaking children see Vidović Zorić & Blažeković, 2023; for English-speaking children see Suh et al., 2014) or higher production of filled pauses in autistic individuals (for children in  Japanese, see Tanaka et al., 2014; for adults in Finnish, see Pirinen et al., 2023).

*Silent Pauses:* Morett et al. (2016) discovered that monolingual autistic adolescents exhibited a higher occurrence of silent pauses lasting over 2 s while describing a cartoon task, aligning with the conclusions of Lake et al. (2011) who also utilized a threshold of over 2 s, albeit in conversational tasks involving adults. Vidović Zorić and Blažeković (2023) found that autistic children exhibited a notably higher frequency of silent pauses occurring within phrases and utterances lasting over 120 ms during the retelling of a cartoon task. However, Thurber and Tager-Flusberg (1993) found reduced production of nongrammatical (within phrase)  silent pauses over 250 ms, in autistic children during picture book narrations.

*Self-corrections and Repetitions*: In the same vein, studies examining self-corrections in autistic children show mixed results in comparison to Lake et al. (2011), reporting both lower production of self-corrections (de Marchena & Eigsti, 2016; Suh et al., 2014) or no significant differences (Greco et al., 2023; MacFarlane et al., 2017; Morett et al., 2016; Thurber & Tager-Flusberg, 1993; Vidović Zorić & Blažeković, 2023) between monolingual autistic children and non-autistic counterparts. Likewise, the available evidence regarding repetitions yielded mixed results, with some studies indicating an increased production of repetitions in autistic children (Kuijper et al., 2017; Suh et al., 2014) or no significant differences in repetitions between autistic and non-autistic children (Greco et al., 2023; MacFarlane et al., 2017; Morett et al., 2016; Thurber & Tager-Flusberg, 1993; Vidović Zorić & Blažeković, 2023).

*Prolongations:* No significant differences in prolongations between autistic and non-autistic children were observed in Vidović Zorić and Blažeković (2023). Wiklund and Laakso (2021) in their study on 11–13-year-old Finnish-speaking boys, reported that disfluencies produced by autistic preadolescents seem to reflect the speaker’s grammatically disturbed speech processing. In contrast, the disfluencies, which included mainly filler words such as *like* and *well*, filled pauses and prolongations, produced by non-autistic preadolescents were characterized by the researchers as more listener-oriented. Conversely, prolongations, observed at a higher frequency in French-speaking autistic adults, were categorized as speaker-oriented disfluencies. This classification was corroborated by variations in conditions involving differing levels of social interaction, such as direct versus averted gaze from the examiner (for French-speaking individuals, see Clin & Kissine, 2023).

### The Link Between Disfluencies and Pragmatic Language

The groundbreaking research by Hallin et al. (2016) proposed to assess correlations between filled pauses and social-pragmatic measures. Irvine et al. (2016) and Hallin et al. (2016) identified an adverse association between social-pragmatic measures and the presence of filled pauses. On the other hand, MacFarlane et al. (2017), McGregor and Hadden (2020), and Lawley et al. (2022) did not find significant links between filled pauses or the proportion of content disfluencies (those occurring with content words) and social-pragmatic measures. However, MacFarlane et al. (2017), McGregor and Hadden (2020), and Lawley et al. (2022) documented no links between filled pauses or the proportion of content disfluencies (those including content words) and social or pragmatic measures. However, Gorman et al. (2016) noted mild negative correlations between social communication capabilities and the ratio of filled pauses. In Dutch-speaking children across all three groups—autistic, non-autistic, and non-autistic with ADHD—Kuijper et al. (2017) discovered a correlation between CCC-2 General Pragmatic Score and speech fluency particularly in the autistic group, and not in the two other groups. Notably, Kuijper et al. (2017) also examined the correlation of ToM with speech fluency and found that children with higher scores on ToM exhibited fewer speech fluency problems and scored higher on discourse pragmatic measures. The discrepancies among these findings may stem from variations in how pragmatic skills were assessed in different studies using different caregiver-derived measures of social communication, such as the Children’s Communication Checklist-2 (CCC-2; Bishop, 2003), the Social Communication Questionnaire (SCQ; Rutter et al., 2003), Autism Diagnostic Interview-Revised (ADI-R; Lord et al., 1994), Social Responsiveness Scale (SRS; Constantino & Gruber, 2005). Additionally, some studies employed clinician-based measures, such as the Autism Diagnostic Observation Schedule, Second Edition (ADOS-2; Lord et al., 2012).

### The Production of Disfluencies in Bilingual Non-Autistic Children

Cross-linguistic studies on the production of disfluencies in bilingual children conflict in terms of overall disfluency rates and types. Studies comparing bilingual and monolingual children have revealed increased overall disfluency rates in bilinguals (Arslan et al., 2023; Byrd et al., 2015; Eggers et al., 2020); however, some investigations have found no noticeable distinctions between the two groups (Bedore et al., 2006; Fiestas et al., 2005).

In the realm of overall disfluency rates across the two languages of bilinguals, Eggers et al. (2020) identified a higher overall disfluency rate in the non-dominant Dutch language of bilingual Yiddish-Dutch-speaking children during conversational tasks. Brundage and Rowe (2018) observed that 30-month-old simultaneous Spanish–English bilinguals exhibited higher overall disfluency rates in SL-English compared to HL-Spanish during conversational tasks. Byrd et al. (2015) noted higher overall disfluency rates in HL-Spanish among Spanish–English bilinguals during narrative tasks, irrespective of language dominance. Fichman and Altman (2023) reported comparable overall disfluency rates in both the HL-Russian and SL-Hebrew among bilingual children, with and without Developmental Language Disorder (DLD), in narrative tasks.

Concerning types of disfluencies produced by bilinguals, some studies reported no differences in the distribution of disfluencies between languages, while others found variations. For example, Byrd et al. (2015) observed higher frequencies of repetitions and grammatical self-corrections in HL-Spanish, but more lexical self-corrections in SL-English. Fichman and Altman (2023) discovered more silent pauses in HL-Russian compared to SL-Hebrew in both non-autistic and DLD groups. Finally, Arslan et al. (2023) detected more silent pauses and repetitions in the non-dominant English language than in the native Turkish language of bilingual Turkish-English-speaking children regardless of age (the sample included 5-year-old bilingual children, that were already exposed to English in English immersion preschool for 3 years and 7-year-old children who had completed 3 years of education in the same school and continued their primary school education in Turkish for 2 years.

The production of disfluencies in children appears to be dynamic across the years. For instance, 7-year-old Turkish-English bilinguals produced more filled pauses than 5-year-old bilinguals regardless of language (Arslan et al., 2023). Martinez-Nieto et al. (2023), comparing production patterns of disfluencies in bilingual Spanish–English speaking children with DLD and bilingual non-autistic Spanish–English speaking children throughout 5 years, reported that the control group showed a gradual increase in overall disfluency percentage, while the DLD group displayed the opposite trend. Moreover, both groups showed a decrease in repetitions and filled pauses in first grade, followed by an increase in these disfluencies by third grade.

## Research Questions

As outlined in the introductory subsection, past studies comparing the production of disfluency between autistic and non-autistic children have consistently shown a lower frequency of filled pauses, but less consistent results were found for repetitions, self-corrections, silent pauses, and prolongations. Yet, it remains uncertain whether bilingual autistic and non-autistic children also produce filled pauses and other disfluencies differently. Therefore, this study aimed to compare the production of disfluencies in autistic vs. non-autistic groups in their two languages (HL-Russian and SL-Hebrew). The study aimed to address the following research questions:

- Research Question 1 (RQ1): Do bilingual autistic children differ from their bilingual non-autistic peers in the production of disfluencies in their two languages (HL-Russian vs. SL-Hebrew)? Based on the results of RQ1, we aim to explore the listener-oriented vs. speaker-oriented status of disfluencies in the bilingual context, building on the findings from previous studies (Clin & Kissine, 2023; Gorman et al., 2016; Hallin et al., 2016; Irvine et al., 2016; Lake et al., 2011; Lawley et al., 2022; MacFarlane et al., 2017; McGregor & Hadden, 2020).
- Research Question 2 (RQ2): Do bilingual autistic children and their non-autistic peers differ in the production of disfluencies across their two languages (HL-Russian vs. SL-Hebrew)? Based on the findings of RQ2, we aim to discuss language-specific vs. language-universal aspects of disfluency production in speech, considering the typological differences between HL-Russian and SL-Hebrew.
- Research Question 3 (RQ3): To what extent are disfluencies linked to language, cognitive, and mentalizing, i.e., ToM skills in both groups, as well as to the *Autism Diagnostic Observation Schedule* scores (ADOS; Lord et al. 2012) in the autistic group. The findings from (RQ3) are anticipated to offer an understanding of the underlying mechanisms behind the occurrence of disfluencies in bilingual autistic and non-autistic children.

## Methods

The materials, data, and analysis script for this study can be retrieved from: https://osf.io/256t4/?view_only=9b9948fe0a2c45a4b33da497ee90d237

### Participants

Participant characteristics are provided in Table 1. The sample of the current study largely overlaps with the sample in Meir and Novogrodsky (2019; 2020; 2023), which examined the language, cognitive, and mentalizing abilities of bilingual and monolingual autistic and non-autistic children. A total of 51 bilingual Russian-Hebrew-speaking children aged 5–9 residing in Israel participated in the current study, divided into two groups: (1) bilingual non-autistic typically developing children, hereafter BI-TD (*n* = 30, 16 girls and 14 boys, Mean age (SD): 6.7 (1.1)) and (2) bilingual autistic children, hereafter BI-ASD (*n* = 21, 5 girls and 16 boys, Mean age (SD): 7.3 (1.3)). The independent t-tests did not reveal any notable discrepancies between the groups concerning Age, Age of Onset of Bilingualism (AOB), SL-Hebrew Length of Exposure (LoE), and Current Exposure to SL-Hebrew (refer to Table 1 for details). However, a notable socioeconomic status (SES) contrast emerged between the autistic and non-autistic groups. Mothers within the autistic group demonstrated significantly fewer years of education compared to those in the non-autistic cohort (*p* = 0.02). Moreover, there was a slight variance in the gender distribution between the two groups, with fewer females observed in the BI-ASD group relative to the BI-TD group (*p* = 0.07), aligning with the established male predominance in ASD diagnoses (Loomes et al., 2017).

**Table 1:** Demographic and background information (M, SD, range) for the entire sample, autistic (BI-ASD) and non-autistic (BI-TD) bilingual groups

	BI-ASD (*n* = 21) N or M (SD)[range]	BI-TD (*n* = 30) N or M (SD)[range]	Between-group differences
Age at first testing (years)	7;3 (1;2) [5–9]	6;7 (1;1) [5–9]	*t* (49) = 1.71, *p* = 0.09
Gender (girls/boys)	5/16	16/14	*χ2* (1) = 3.31, *p* = 0.07
SES (years of)	15 (3.6) [11–25]	18 (3.3) [10–24]	*t* (49) = − 2.47, *p* = **0.02**
AOB (months of)	26 (24) [0–80]	16 (18) [0–48]	*t* (49) = 1.66, *p* = 0.10
LoE (months of)	61 (22) [19–108]	64 (20) [35–96]	*t* (49) = − 0.91, *p* = 0.63
Current exposure (%)	57 (14) [25–75]	52 (14) [25–75]	*t* (49) = 1.07, *p* = 0.29

Groups were compared using independent *t*-tests, except for the Gender ratio, where a Chi-square test was employed

Data are presented as means, standard deviations, and ranges

*BI-TD* Bilingual non-autistic children,* BI-ASD* Bilingual autistic children, *SES* Socio-economic status, measured by maternal education in years, *AOB* age of onset of bilingualism, *LoE* length of exposure to SL-Hebrew

Current Exposure to SL-Hebrew: The amount of linguistic input in SL-Hebrew

Significant differences (*p* < 0.05) are highlighted in bold font

### Recruitment and Consent

The participant selection process in this study was guided by the utilization of the Bilingual Parents' Questionnaire (BIPAQ) and interviews (Abutbul-Oz & Armon-Lotem, 2022). The BIPAQ elicited background and language use/exposure information for all children in the current study. In the context of this research, bilingual children were identified as those who: (i) Acquired Russian as their home language through interaction with parent(s) who are Russian speakers; (ii) Had been exposed to two languages for a minimum of 9 months before participating in the study and demonstrated proficiency in using both languages.

The non-autistic group was recruited from mainstream kindergartens and schools, while the autistic group was drawn from diverse educational settings, including ASD classes, special education, and/or mainstream schools. For inclusion in the non-autistic group, participants needed to meet specific criteria: (i) No expression of parental concerns regarding their children's language milestones; and (ii) No reported developmental disorders, encompassing ASD, DLD, ADHD, or hearing impairments, as confirmed by parental responses.

Participants with ASD were included based on formal diagnoses provided by a psychiatrist or psychologist, aligning with established criteria in Israel. The diagnostic status of autistic children was verified using the Autism Diagnostic Observation Schedule, Second Edition (ADOS-2, Lord et al., 2012) as part of the study battery.

### Procedures

The study was authorized by the IRB of Bar-Ilan University and by the Chief Scientist of the Israel Ministry of Education. The study procedures for all participants were identical and followed the protocols used in previous studies at Bar-Ilan University. Before the researchers met with each child, parents were given a brief explanation of the study, both verbally and in detailed written form in Russian and Hebrew. All parents signed a consent form before the first meeting. The study tasks were conducted in the participants' homes or at their schools at various times of the day, based on the participants' availability, preferences, and needs. Testing was divided into three to five sessions, each lasting approximately 30–60 min (depending on the child's cooperation), conducted in both HL-Russian and SL-Hebrew. The tasks were presented in a randomized order across participants. Before each meeting, the child's oral assent was obtained. All sessions were audio-recorded for further offline analysis.

### Language and Cognitive Assessment Measures

Due to the large number of measures included in this study, only brief descriptions are provided for the language and cognitive assessment control measures. Additional details are available upon request from the author and/or in previous studies that describe the current sample (Meir & Novogrodsky, 2019, 2020, 2023).

- The Autism Diagnostic Observation Schedule (ADOS; Lord et al., 2012) is a semi-structured play-based assessment and is the gold-standard tool for diagnosing ASD.
- The verbal ToM battery was used to assess the mentalizing ability to attribute mental states to oneself and others. It included three verbal *ToM* tasks: a content false-belief task (‘Smarties task’) (the unexpected content, Perner et al., 1987). For the first-order false-belief task and second-order false-belief (Baron-Cohen et al., 1985; Wimmer & Perner, 1983), computerized film versions of the tasks (Buac and Kaushanskaya, 2020) were adapted from English to Hebrew.
- The Hebrew Forward Digit Span (FWD) and the Hebrew Backward Digit Span (BWD) adapted from the Wechsler Intelligence Scale for Children (Wechsler, 1991) were used to assess verbal short-term memory (vSTM) and verbal working memory (vWM). The Raven's Colored Progressive Matrices (Raven, 1998) were utilized to assess the Nonverbal Intelligence Quotient (NVIQ).

For language assessment in SL-Hebrew and HL-Russian, this study made use of the Language Impairments Testing in Multilingual Settings (LITMUS) tools developed within the CO Action IS0804 project titled ‘Language Impairment in a Multilingual Society: Linguistic Patterns and the Road to Assessment’.

- Hebrew and Russian LITMUS Sentence Repetition tasks (SRep-30), based on SRep 56 tasks (Armon-Lotem & Meir, 2016; Meir et al., 2016), were administered to assess morpho-syntactic abilities. The tasks in both languages contained 30 sentences of varying morpho-syntactic complexity previously reported to be challenging to children with language impairments (e.g., relative clauses, direct and indirect object questions).
- Cross-linguistic Lexical Tasks (LITMUS-CLT, henceforth CLT) were administered to assess both receptive and expressive knowledge of nouns and verbs using the Hebrew CLT task (Altman et al., 2017) and the Russian CLT task (Gagarina & Nenonen, 2017). Each picture naming and picture choice task in each language was composed of 32 items (nouns and verbs).

We compared the cognitive and mentalizing measures, conducted in SL-Hebrew of the two bilingual groups (autistic and non-autistic) using independent *t*-tests (Online Resource 2) Independent *t*-tests showed that autistic children did not differ in performance on cognitive measures compared to their non-autistic peers. However, in terms of mentalizing measures (ToM), the autistic group scored significantly lower than the non-autistic group (*t* (49) =  − 2.5, *p* = 0.02).

We conducted independent* t*-tests to compare the Language Assessment measures between the two bilingual groups (autistic and non-autistic) in both languages (Online Resource 3). Results from independent* t*-tests revealed that non-autistic children demonstrated significantly higher performance compared to autistic children on SRep-30 in SL-Hebrew (*t* (28.61) =  − 3.85, *p* = 0.0006) as well as in HL-Russian (*t* (23.16) =  − 2.11, *p* = 0.045). Additionally, in SL-Hebrew, non-autistic children outperformed autistic children on CLT-Verb Receptive (*t* (49) =  − 2.66, *p* = 0.010) and CLT-Verb Production (*t* (47) =  − 2.62, *p* = 0.012). However, there were no significant differences between the two groups in performance on other CLT tasks.

### Experimental Task

#### Stimuli

In this study, narratives were elicited using the LITMUS Multilingual Assessment Instrument for Narratives (MAIN, Gagarina et al., 2019), designed to assess the narrative skills of bilingual children aged 3–10, both with and without diagnosed language impairment, for various languages and language combinations. For the current study, the storytelling mode was employed. The HL-Russian narrative assessment utilized the Baby Goats story, while the SL-Hebrew narrative assessment used the Baby Birds story. Following the instructions for the telling mode (Gagarina et al., 2019), the researcher sat opposite the child, allowing the child to hold the pictures facing towards them, but away from the experimenter. The researcher then said, *‘Look, here are 3 envelopes. There is a different story in each envelope. Choose one, and then you can tell me a story*.’ When the child took out the pictures, the researcher asked her/him to unfold the images and look at the whole story starting with the first picture, without showing the pictures to the researcher. Afterward, the researcher assisted the child in unfolding the first two pictures and asked them to tell the best story they could. Upon completion of the first two pictures, the researcher helped the child unfold the next two pictures, allowing all four pictures to be seen and repeated the process until the story concluded.

### Transcription and Coding Conventions

The audio-recorded narrations, including all disfluencies, were transcribed using the *Codes for the Human Analysis of Transcripts* (CHAT) format, which enables coding to be performed using the *Computerized Language Analysis* (CLAN) program (MacWhinney, 2000). All disfluencies were manually segmented by a Russian-Hebrew speaker Speech and Language Pathologist (the first author). For HL-Russian and SL-Hebrew narratives, we utilized a transliteration method based on the *Speech Assessment Methods Phonetic Alphabet* (SAMPA).

We employed the 'FREQ' function of the Computerized Language Analysis (CLAN) to generate a list of word tokens (total number of words) and word types (total number of different words). Extraneous comments unrelated to the story and disfluencies were excluded from the analysis. All narratives were analyzed for productivity, including the number of word tokens, word types and conversational units (c-units), wherein each c-unit contained one independent clause and all its dependent clauses (MacWhinney, 2000). Error ratio included both morpho-syntactic errors (e.g., errors in number, case, definiteness, and gender) and lexical-semantic errors.

We examined 11 types of disfluencies in both languages, based on CHAT categorization and transcription, with special characters used to mark disfluencies, along with some adaptations for our study (Online Resource 4). Our list of disfluencies included silent pauses (intra-utterance silent pauses) filled pauses, polysyllabic whole-word repetitions, monosyllabic whole-word repetitions, phrase repetitions, word self-corrections, phrase self-corrections, phonological fragments, part-word repetitions, prolongations, and broken words. The  disfluency  rate, a measure that quantifies the occurrence of disfluencies in speech, was computed based on the frequency of different types of disfluencies within a given speech sample. To control for narrative length differences, disfluency rates were generated by dividing the total number of each disfluency type by the word tokens in the narrative (excluding words in disfluencies).

#### Statistical Analysis

To compare the general narrative measures, we constructed four separate linear mixed-effects models for c-units, word tokens, word types and error ratio as dependent variables with Group (autistic: BI-ASD, non-autistic: BI-TD) and Language (HL-Russian, SL-Hebrew) as fixed effects, and participant as a random effect. The analysis was performed using *lme4* package (Bates et al., 2020)  in R  (R Core Team, 2020).

To address our research questions RQ1 and RQ2, we utilized a linear mixed-effects model using *lme4* package (Bates et al., 2020) for all hypothesis testing.  We fitted the model for  disfluency  rate with Age, Group (autistic: BI-ASD, non-autistic: BI-TD), Language (HL-Russian vs. SL-Hebrew), and Disfluency Type (11 types of disfluencies) as fixed effects, and participant as a random effect. Marginal R squared was calculated as a measure of model fit for fixed effects, and conditional R squared was used where appropriate for fixed and random effects. For interactions, pairwise comparisons were conducted using the *emmeans* function of the *emmean*s library (Lenths, 2022), and for these, we provided the estimate, SE,* t*.ratio, and *p*-values.

For RQ3, which aimed to explore the relationship between disfluencies and various demographic, background, language, cognitive, and general narrative measures, we conducted Spearman correlation analysis in R using the *tab_corr* function from *sjplot * package (Lüdecke, 2021).

## Results

### General Narrative Measures

The results in four linear mixed models revealed that both the non-autistic and autistic groups told stories of comparable productivity, as measured by a number of c-units and word tokens, and of comparable lexical diversity, as measured by a number of word types (Table 2 and Online Resource 5). However, for the error ratio, both the Group and Language effects were statistically significant (*p* = 0.026 and *p* = 0.011, respectively). Pairwise comparisons further showed that the autistic group exhibited a higher error ratio than non-autistic (*p* = 0.0096), and that errors were more prevalent in HL-Russian than in SL-Hebrew for both groups (*p* = 0.0002).

**Table 2:** Comparative analysis of general narrative measures between BI-ASD and BI-TD groups in each language

	HL-Russian		SL-Hebrew		Statistical analysis					
	BI-ASD (*n* = 20)	BI-TD (*n* = 28)	BI-ASD (*n* = 20)	BI-TD (*n* = 27)	Main effects and interactions	EST	*t*	*p*	Contrast (emmeans,SE)	*p*
C-units	9.70 (2.64)	10.14 (2.82)	9.80 (3.17)	10.59 (2.71)	Group Language Group:Language	0.82 − 4.13e − 03 − 0.33	0.98 − 6.26e − − 0.38	0.331 0.995 0.707		
Tokens	48.30 (20.31)	48.64 (24.51)	57.25 (22.93)	65.81 (27.94)	Group Language Group:Language	8.47 − 7.80 − 7.59	1.18 − 1.78 − 1.31	0.240 0.079 0.193		
Types	29.50 (11.96)	29.29 (12.01)	32.20 (10.48)	32.37 (9.88)	Group Language Group:Language	0.12 − 2.30 − 0.28	0.04 − 1.00 − 0.09	0.970 0.322 0.927		
Error ratio	0.25 (0.21)	0.16 (0.10)	0.16 (0.13)	0.08 (0.07)	Group Language Group:Language	− 0.09 0.08 1.60e − 03	− 2.26 2.61 0.04	**0.026** **0.011** 0.970	BI-ASD (0.206, 0.0239) > BI-TD (0.121, 0.0204) SL (0.121, 0.0189) < HL (0.205, 0.0188)	**0.0096** **0.0002**

Table 2 compares groups (BI-ASD and BI-TD) and languages (HL-Russian and SL-Hebrew) across narrative measures: c-units, word tokens, word types, and error ratio. Linear mixed-effect models were used for each measure. Data include means, standard deviations, and ranges for each group and language. The statistical analysis includes estimated effect size (*EST*), *t*-value, and *p*-value. Statistically significant *p-*values (*p* < 0.05) are highlighted in bold, with detailed contrasts provided by estimated marginal means (*emmeans*) and standard errors (*SE*).

### The Production of Disfluencies in Bilingual Autistic and Non-Autistic Groups in Both Languages

Research questions RQ1 and RQ2 were designed to explore differences in the production of disfluencies  across two contexts: RQ1 investigates differences between autistic and non-autistic groups within each language while RQ2 examines differences between HL-Russian and SL-Hebrew within each group.

To address both research questions, we fitted the linear mixed model to predict disfluency rate with Age, Group (autistic: BI-ASD, non-autistic: BI-TD), Language (HL-Russian vs. SL-Hebrew), and Disfluency Type (eleven types of disfluencies) (see Table 3). The model's total explanatory power is moderate (overall model: conditional *R*2 = 0.23; and the part related to the fixed effects alone marginal *R*2 = 0.16).

**Table 3:** Final model for disfluency production

Predictors	Estimates	SE	CI	Statistic	*p*
(Intercept)	0.02	0.01	− 0.01 to 0.04	1.45	0.146
Age	0.00	0.00	− 0.00 to 0.00	0.40	0.693
Group [BI-TD]	0.06	0.01	0.04 to 0.08	6.04	** < 0.001**
Language [SL-Hebrew]	0.01	0.01	− 0.01 to 0.03	0.89	0.375
DisfluencyType [SilentPauses]	− 0.00	0.01	− 0.02 to 0.01	− 0.52	0.607
Group [BI-TD] × Language [SL-Hebrew]	− 0.06	0.01	− 0.09 to − 0.04	− 4.93	** < 0.001**
Group [BI-TD] × DisfluencyType [SilentPauses]	− 0.03	0.01	− 0.05 to − 0.00	− 1.97	**0.049**
Language [SL-Hebrew] × DisfluencyType [SilentPauses]	− 0.01	0.01	− 0.04 to 0.01	− 0.87	0.382
(Group [BI-TD] × Language [SL-Hebrew]) × DisfluencyType [Prolongations]	0.08	0.02	0.04 to 0.11	4.22	** < 0.001**
Random effects					
σ^2^	0.00				
τ_00_ _Code_	0.00				
ICC	0.08				
N _Code_	50				
Observations	1045				
Marginal *R*^2^/Conditional *R*^2^	0.162/0.233				

Table 3 displays estimates, standard errors, and the significance of coefficients in the final model. The groups analyzed are BI-TD (Bilingual non-autistic children) and BI-ASD (Bilingual autistic children), with languages HL-Russian and SL-Hebrew. Disfluency Type encompasses eleven categories: filled pauses, silent pauses, polysyllabic whole-word repetitions, phrase repetitions, whole-word self-corrections, phrase self-corrections, phonological fragments, part-word repetitions, monosyllabic whole-word repetitions, prolongations, and broken words

Note 1: One child from the BI-ASD group and two from the BI-TD group declined to participate in the HL-Russian narrative task. One child from the BI-ASD group and three from the BI-TD group declined to participate in the SL-Hebrew narrative task

Note 2: Significant differences (*p* < 0.05) are highlighted in bold font

**Fig. 1:**
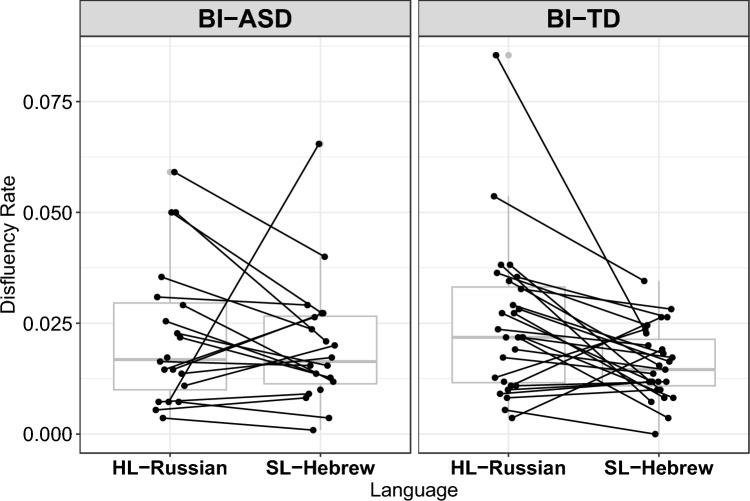
Comparison of disfluency rate per language (HL-Russian vs. SL-Hebrew) per group (autistic: BI-ASD; non-autistic: BI-TD). Boxplot showing the distribution of Disfluency Rate across different Languages for two groups: BI-ASD (Bilingual Autistic Children Group) and BI-TD (Bilingual non-autistic children Group). *X*-axis (Language): Represents the languages used, where HL stands for Russian (the home language) and SL stands for Hebrew (the societal language). *Y*-axis (disfluency rate): Indicates the rate of disfluencies observed in the speech of participants, measured as the number of disfluencies per word token. Facet Grid (Group): Separates the data into two facets based on the group, with one facet for BI-ASD (Bilingual autistic group) and another for BI-TD (Bilingual non-autistic group)

**Fig. 2:**
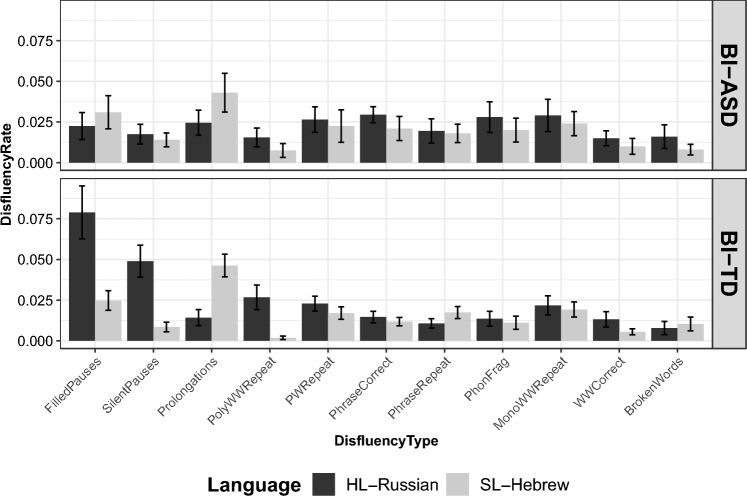
Disfluency rate distribution per disfluency type per language per group (bilingual autistic: BI-ASD; bilingual non-autistic: BI-TD). Bar plot illustrates  the disfluency rate for different Disfluency Types across Languages for two groups: BI-ASD (Bilingual autistic group) and BI-TD (Bilingual non-autistic group). Error bars represent standard errors. *X*-axis (Disfluency Type): Represents 11 types of disfluencies observed in speech. *Y*-axis (Disfluency Rate): Indicates the rate of occurrence of disfluencies, measured as the number of occurrences per word token. Facet Grid (Group): Separates the data into two facets based on the group, with one facet for the BI-ASD group and another for the BI-TD group. Note: Different fill colors represent different languages

Within the final model, the impact of the Group (autistic vs. non-autistic) on disfluency rate was statistically significant (*EST* = 0.06, *t*(998) = 6.04, *p* < 0.001), indicating that the non-autistic group produced higher overall disfluency rates compared to the autistic group (Fig. 1). In addition, a significant two-way Group*Language interaction was detected (Fig. 1). For non-autistic children, the contrast between HL-Russian and SL-Hebrew revealed a significant difference (*p* = 0.0003), with higher disfluency rates observed in their HL-Russian compared to SL-Hebrew. Figure 2 highlights how disfluency rate vary across different disfluencies and languages within each group. In addition, it provides insights into the prevalence and distribution of specific types of disfluencies in different linguistic contexts.

#### RQ1: Between-Group Comparison of Disfluencies

In the final model, three-way Group*Language*Disfluency Type interactions were observed (Table 3 and Fig. 2) suggesting that Disfluency Type rates varied across the groups and the two languages of bilinguals. The results of pairwise comparisons focusing on Group differences within the three-way Group*Language*Disfluency Type interaction in the disfluency production demonstrated that the autistic group exhibited a significantly lower disfluency rate than the non-autistic group for both filled pauses (*p* < 0.001) and silent pauses (*p* = 0.001) (see Table 4).

**Table 4:** Pairwise disfluency type comparisons across the two languages (HL-Russian vs. SL-Hebrew with the contrast of Group (bilingual autistic: BI-ASD; bilingual non-autistic: BI-TD)

	Disfluency type	Estimate	SE	*t*.ratio	*p*-value
HL-Russian	FilledPauses	0.056	0.009	− 6.043	** < 0.0001**
	SilentPauses	0.032	0.009	− 3.390	**0.001**
	Prolongations	0.010	0.009	1.031	0.303
	PolyWWRepeat	0.012	0.009	− 1.251	0.211
	PWRepeat	0.003	0.009	0.333	0.739
	PhraseCorrect	0.014	0.009	1.523	0.128
	PhraseRepeat	0.008	0.009	0.879	0.380
	PhonFrag	0.014	0.009	1.478	0.140
	MonoWWRepeat	0.007	0.009	0.712	0.477
	WWCorrect	0.001	0.009	0.136	0.892
	BrokenWords	0.008	0.009	0.811	0.418
SL-Hebrew	FilledPauses	0.006	0.009	0.638	0.524
	SilentPauses	0.005	0.009	0.564	0.573
	Prolongations	− 0.003	0.009	− 0.361	0.718
	PolyWWRepeat	0.006	0.009	0.581	0.561
	PWRepeat	0.006	0.009	0.562	0.574
	PhraseCorrect	0.009	0.009	0.950	0.342
	PhraseRepeat	0.000	0.009	0.049	0.961
	PhonFrag	0.009	0.009	0.923	0.356
	MonoWWRepeat	0.005	0.009	0.486	0.627
	WWCorrect	0.004	0.009	0.455	0.650
	BrokenWords	− 0.003	0.009	− 0.264	0.792

Table 4 presents the estimates, standard errors, *t*-ratios, and *p*-values for the contrasts between the BI-ASD (Bilingual autistic group) and BI-TD (Bilingual non-autistic group) within each Disfluency Type and Language (HL-Russian vs. SL-Hebrew). Pairwise comparisons were conducted utilizing the emmeans package

Note: Significant differences (*p* < 0.05) between groups (bilingual autistic (BI-ASD) vs. bilingual non-autistic (BI-TD) are highlighted in bold font

### RQ2 Within-Group Comparisons of Disfluencies

The results of pairwise comparisons focusing on Language differences within the three-way Group*Language*Disfluency Type (Table 3, Fig. 2) interaction in the disfluency production demonstrated that the non-autistic group exhibited significantly higher rates for filled pauses (*p* < 0.0001), silent pauses (*p* = 00001) and polysyllabic whole word repetitions in HL-Russian (*p* = 0.003) than in SL-Hebrew and a significantly higher rate for prolongations in SL-Hebrew than in HL-Russian (*p* = 0.000) (see Table 5).

**Table 5:** Pairwise disfluency type comparisons per Group (bilingual autistic: BI-ASD; bilingual non-autistic: BI-TD) with the contrast of Language (HL-Russian vs. SL-Hebrew)

	Disfluency type	Estimate	SE	*t* ratio	*p*-value
BI-ASD	FilledPauses	− 0.009	0.010	− 0.888	0.375
	SilentPauses	0.003	0.010	0.346	0.729
	Prolongations	− 0.019	0.010	− 1.917	0.056
	PolyWWRepeat	0.008	0.010	0.809	0.418
	PWRepeat	0.004	0.010	0.398	0.691
	PhraseCorrect	0.008	0.010	0.861	0.390
	PhraseRepeat	0.001	0.010	0.141	0.888
	PhonFrag	0.008	0.010	0.809	0.418
	MonoWWRepeat	0.005	0.010	0.501	0.617
	WWCorrect	0.005	0.010	0.501	0.617
	BrokenWords	0.008	0.010	0.809	0.418
BI-TD	FilledPauses	0.054	0.008	6.556	** < 0.0001**
	SilentPauses	0.041	0.008	4.903	** < 0.0001**
	Prolongations	− 0.032	0.008	− 3.832	**0.000**
	PolyWWRepeat	0.025	0.008	3.037	**0.003**
	PWRepeat	0.006	0.008	0.731	0.465
	PhraseCorrect	0.003	0.008	0.366	0.715
	PhraseRepeat	− 0.006	0.008	− 0.778	0.437
	PhonFrag	0.003	0.008	0.326	0.745
	MonoWWRepeat	0.003	0.008	0.334	0.739
	WWCorrect	0.008	0.008	0.953	0.341
	BrokenWords	− 0.002	0.008	− 0.274	0.784

Table 5 presents the estimates, standard errors, *t*-ratios, and *p*-values for the contrasts between HL-Russian and SL-Hebrew within each Disfluency Type and Group BI-ASD: Bilingual autistic group and BI-TD: Bilingual non-autistic group. Pairwise comparisons were conducted using the emmeans package

Note: Significant differences (*p* < 0.05) between languages (HL-Russian vs. SL-Hebrew) are highlighted in bold font

### RQ3 Relationship Between Disfluencies and Demographic and Narrative Variables

In our  third research question (RQ3) we aimed to examine the relationship between disfluencies and demographic, background, language, cognitive, and general narrative measures to better understand underlying mechanisms behind the production patterns and distribution of disfluencies. Correlational analysis was conducted for each group in each language, examining the relationships between disfluencies and various variables (Chronological Age, AOB, Hebrew and Russian CLT-Verb Receptive subtasks, Hebrew and Russian SRep-30 tasks, FWD, BWD, NVIQ, ToM, and Error ratio) using Spearman correlation coefficients. Disfluencies identified as significant in between-group (autistic vs. non-autistic) or within-group (HL-Russian vs. SL-Hebrew) comparisons, such as silent pauses, filled pauses, polysyllabic whole-word repetitions, and prolongations, were included in the analysis.

For the non-autistic group in HL-Russian, the results of the correlational analysis demonstrated a strong positive correlation between prolongations and filled pauses (*r* = 0.62, *p* < 0.001) (Online Resource 6). Concerning silent pauses produced in HL-Russian by the non-autistic group, there was a positive correlation between silent pauses and BWD (*r* = 0.41, *p* = 0.03) indicating that non-autistic children with better working memory abilities tend to produce more silent pauses in HL-Russian. Polysyllabic whole-word repetitions correlated negatively with ToM scores in the non-autistic group (*r* =  − 0.43, *p* = 0.02) indicating that non-autistic children with better mentalizing abilities tend to produce fewer polysyllabic whole-word repetitions in HL-Russian. In the non-autistic group, there was a negative correlation between SES as measured by maternal education in years and error ratio (*r* = 0.46, *p* = 0.01). This suggests that non-autistic children with more errors in their HL-Russian narratives tend to come from higher SES backgrounds, characterized by mothers with more years of education.

In SL-Hebrew for the non-autistic group (Online Resource 7), the results revealed that silent pauses correlated positively with several variables: Age (*r* = 0.61, *p* < 0.001); AOB (*r* = 0.67, *p* < 0.001); NVIQ (*r* = 0.59, *p* < 0.001). There were no correlations between SES and any of the variables for the non-autistic group in SL-Hebrew.

As for the autistic group, the results in HL-Russian showed a positive correlation between the CLT-Verb Receptive in HL-Russian and ToM (*r* = 0.47, *p* = 0.03) and a negative correlation between error ratio and ToM (*r* =  − 0.71, *p* =  < 0.001) indicating that autistic children with better mentalizing abilities tend to have higher lexical abilities (Online Resource 8). In SL-Hebrew for the autistic group (Online Resource 9), similar to results in HL-Russian, there was a positive correlation between ToM and CLT-Verb Receptive (*r* = 0.84, *p* < 0.001) and a negative correlation between ToM and error ratio (*r* =  − 0.60, *p* < 0.001). Furthermore, there was a correlation between ToM and SRep-30 in SL-Hebrew (*r* = 0.85, *p* < 0.001) within the autistic group. These findings across HL-Russian and SL-Hebrew suggest a potential association between heightened mentalizing abilities and linguistic proficiency in bilingual autistic children.

Positive correlations were observed between filled pauses and cognitive abilities, measured by the FWD test (*r* = 0.50, *p* = 0.02) and the BWD test (*r* = 0.49, *p* = 0.02), within the autistic group in SL-Hebrew. These findings suggest a potential association between filled pauses and enhanced cognitive skills among bilingual autistic children, particularly within the context of SL-Hebrew (Online Resource 8). Furthermore, a positive correlation was observed between ToM and filled pauses in HL-Hebrew for the autistic group (*r* = 0.46, *p* = 0.03). These correlations suggest a potential association between filled pauses and enhanced mentalizing skills among bilingual autistic children, particularly within the context of HL-Hebrew (Online Resource 8). No correlations of ADOS with disfluencies were found in the autistic group in both languages.

To explore the consistency of the relationship between filled pauses and ToM across both languages, we conducted additional correlation analyses separately for both the autistic and non-autistic groups, disregarding language differences (Online Resource 10 for autistic and Online Resource 11 for non-autistic children). The analyses unveiled a noteworthy significant correlation between filled pauses and ToM within the autistic group (*r* = 0.36, *p* = 0.02), whereas no similar association was identified for other types of disfluencies. Moreover, no significant relationship was found between ADOS scores and any of the disfluency types within this group (Online Resource 10). Conversely, in the non-autistic group, there was no apparent relationship observed between ToM and any of the eleven disfluency types (Online Resource 11).

## Discussion

In this study, we investigated disfluencies in bilingual autistic and non-autistic children, aiming to replicate and expand upon prior research conducted on monolingual individuals. For these purposes, we evaluated disfluency production in Russian-Hebrew bilingual autistic (BI-ASD) and non-autistic (BI-TD) children in both languages.

Our first research question examined differences in disfluency production patterns between bilingual autistic and non-autistic children, shedding light on the status of listener- vs. speaker-oriented disfluencies, following Clin and Kissine (2023). The results regarding between-group differences in disfluency types production confirmed that bilingual autistic children produced filled pauses less frequently than their non-autistic peers, consistent with Clin and Kissine (2023) and with previous studies in monolingual children (Gorman et al., 2016; Hallin et al., 2016; Heeman et al., 2010; Irvine et al., 2016; Jones et al., 2022; Lawley et al., 2022; Lunsford et al., 2010; MacFarlane et al., 2017; McGregor & Hadden, 2020; Morett et al., 2016; Parish-Morris et al., 2017; Salem et al., 2021). Moreover, while the observed correlation between ToM and filled pauses in bilingual autistic children hints at a possible link between enhanced mentalizing skills and greater production of filled pauses, additional evidence and analysis are necessary to firmly establish a pragmatic interpretation.

Differences between groups were noted in the frequency of intra-utterance silent pauses, with bilingual non-autistic children displaying a higher rate in HL-Russian. This aligns with Thurber and Tager-Flusberg’s (1993) findings, which observed fewer intra-phrase silent pauses in monolingual autistic children compared to their non-autistic peers during storytelling task from wordless picture book. However, our findings diverge from Vidović Zorić and Blažeković’s (2023) research, which reported more silent pauses within phrases and utterances produced by monolingual autistic children during a cartoon retelling task. It is important to note that direct comparisons are limited due to variations in the thresholds for defining silent pauses across these studies. Indeed, it seems that within the realm of silent pauses, their duration and turn-taking gaps hold great significance for discerning differences between autistic and non-autistic individuals (Heeman et al., 2010; Tanaka et al., 2014). In analyzing Russian monologues, Korotaev et al. (2020) suggested that sometimes silent pauses, when occur in an “unexpected” position and/or last for an “unexpectedly” long duration could be seen as a mechanism for hesitation. Based on Lake et al. (2011) and Clin and Kissine’s (2023) studies and Korotaev et al.'s (2020) perspective, an increased occurrence of intra-utterance silent pauses among bilingual lanon-autistic children in HL-Russian, compared to their autistic peers, might indicate that these pauses, similar to filled pauses, may function as listener-oriented disfluencies. However, it is important to approach this idea cautiously because there are only a few studies available, and their results are not consistent.

Our second research question aimed to explore the differences in disfluency production between the two languages by autistic and non-autistic groups. We observed that the overall disfluency rate differed between SL-Hebrew and HL-Russian in the non-autistic group with higher rates observed in their HL-Russian compared to SL-Hebrew in line with prior studies that found higher overall disfluency rate in the less dominant language in non-autistic bilingual children (see Rojas et al., 2023).

Specifically, the non-autistic group produced more silent pauses, filled pauses, polysyllabic whole-word repetitions, and fewer prolongations in HL-Russian than in SL-Hebrew. The observed variation between filled pauses and prolongations, both being interchangeable disfluencies, may suggest language-specific patterns and tendencies. In Russian, filled pauses are noted as the predominant type of hesitation phenomenon, encompassing filled pauses, prolongations, and other interruptions, as indicated by Verkhodanova et al. (2018). Conversely, prolongations appear to be more prevalent in Hebrew, as suggested by Silber-Varod (2013). Silber-Varod (2013) further observed that prolongations in Hebrew often occurred within conjunctions (e.g., *ve* meaning 'and'), possessive markers (e.g., *shel* meaning 'of'), definite articles (e.g., *ha* meaning 'the'), and other function words like prepositions. Consequently, in Hebrew, prolongations can frequently replace filled pauses, masking them within various monosyllabic function words ending with the sound /e/, such as *ve*: (meaning 'and'), *le*: (meaning 'to'), *be*: (meaning 'in'), *he*: (meaning 'that'), and *kshe*: (meaning 'when'). This substitution effectively converts filled pauses into prolongations. In contrast, Russian lacks the prevalence of the sound /e/ in most function words (e.g., *v* meaning 'in,' *k*' meaning 'to,' *iz* meaning 'from,' *i* meaning 'and,' *s* meaning 'with'), leading speakers to more frequently employ isolated filled pauses near function words.

Another notable disfluency production pattern observed within the non-autistic group in this study was a greater frequency of polysyllabic whole-word repetitions in their HL-Russian compared to their SL-Hebrew, consistent with the findings of Byrd et al. (2015) and Arslan et al. (2023), that observed more repetitions in the weaker languages of bilingual non-autistic children. This discrepancy might be indicative of a heightened degree of linguistic uncertainty within the non-autistic group when speaking in HL-Russian and potentially serve as an additional opportunity for auditory monitoring of their speech output (Fiestas et al., 2005).

The increased production of silent pauses in HL-Russian by the non-autistic group aligns with the results of Arslan et al. (2023), who identified more silent pauses in the non-dominant English language than in native Turkish, attributing this to a strategy that helps the speakers to inhibit unwanted language switching between the two languages and also helps them to harmonize with their listeners. This explanation may also be relevant to our participants, particularly when suppressing SL-Hebrew during narrative production in HL-Russian. The findings regarding silent pauses among non-autistic children offer partial validation to the conclusions of Fichman and Altman (2023). They noted a higher frequency of silent pauses in HL-Russian compared to SL-Hebrew across both groups, irrespective of the presence of DLD. However, their observation of no significant differences in repetitions, self-corrections, or filled pauses between HL-Russian and SL-Hebrew aligns with the results observed in the autistic group within the current study.

In our third research question, we aimed to examine the relationship between disfluencies and demographic, language, cognitive, and narrative variables to better understand the underlying mechanisms for production patterns of disfluencies and their distributions. Evidence from previous research indicates that while linguistic ability plays a role in ToM comprehension, the pragmatic aspect of language, which pertains to non-structural elements, holds greater significance for ToM understanding (Frank, 2018).

Our findings confirm this claim, as we identified a positive correlation between ToM and filled pauses specifically in autistic children, with no such correlation observed in non-autistic children. Additionally, we explored the relationship between filled pauses and ToM regardless of language and consistently found positive correlations between ToM and filled pauses in autistic children. This suggests a potential pragmatic link between enhanced mentalizing skills and increased production of filled pauses. Furthermore, the positive correlation between BWD and filled pauses observed exclusively in the autistic group suggests that the involvement of working memory in producing listener-oriented disfluencies in this group may be linked to enhanced communicative-pragmatic skills. This notion is supported by Kuijper et al. (2017), who discovered a positive correlation between working memory and discourse pragmatics in Dutch-speaking autistic children. Additionally, Donlan and Masters (2000) found that in children with communication disorders, social skills were predicted by short-term memory ability, further supporting the idea of a relationship between working memory and pragmatic skills.

Nevertheless, it is essential to acknowledge that disparities among previous findings regarding the relationship between pragmatic skills and filled pauses may arise from variations in how pragmatic skills were assessed across different studies using different evaluation tools. Furthermore, it is important to recognize the partial overlap between pragmatics and ToM, as emphasized by Bosco et al. (2018). This highlights the necessity for additional research to establish a stronger connection between filled pauses and pragmatic abilities.

In summary, our study confirms that bilingual autistic Russian-Hebrew-speaking children exhibit a reduced occurrence of filled pauses in HL-Russian in comparison to their non-autistic counterparts, replicating previous findings on monolinguals. Moreover, it has been suggested that filled pauses serve as listener-oriented disfluencies, as evidenced by their association with ToM. Conversely, the role of intra-utterance silent pauses warrants deeper investigation, given the limited support from prior studies. Additionally, our results revealed that bilingual autistic children exhibited consistent patterns in disfluency types across languages, suggesting a more language-universal disfluency production, while non-autistic bilingual children displayed more language-specific disfluency production, with distinct disfluency patterns observed in each language.

### Limitations and Future Research

While our study offers valuable insights into the production of disfluencies among bilingual autistic children, it is important to recognize and address certain limitations inherent in our research. In the present study, we opted for a narrative format using wordless picture books. However, conversations or personal narratives, as suggested by Losh and Capps (2003), could be more challenging for the autistic group. Therefore, future research should carry out comparisons of disfluency production across different modes (monologues, conversations) and contexts. Another limitation of this research concerns the interaction dynamics between examiners and participants. Notably, in all studies where autistic children exhibited fewer filled pauses, participants interacted exclusively with either a research assistant or a test administrator. This suggests a potential variation in the frequency of filled pauses when children interact with individuals who are not in positions of authority (Jones et al., 2022).

While adult experimenters in our study ensured their unfamiliarity with the storytelling mode instructions, the availability of the retelling mode option in the LITMUS MAIN battery allows examining attempts to tailor the production of disfluencies to the listener's needs under shared and unshared knowledge conditions (de Marchena & Eigsti, 2016).

Despite our efforts to match groups on demographic factors, there existed a disparity in socioeconomic status, e.g., mother's education. Potential underdiagnosis among children of mothers with lower educational backgrounds may yield differing outcomes, as proposed by Kelly et al. (2019). It is important to note that the production of silent pauses should be further explored, considering factors such as length, position in phrases, and presence in disfluency clusters, which were beyond the scope of this study.

Future studies should further explore how the production of disfluencies is influenced by bilingualism by comparing bilingual autistic children to their monolingual counterparts in both languages. Moreover, an exploration of disfluencies in bilingual autistic and non-autistic children who speak diverse language pairs, across a range of speech modes including personal narratives and conversations, could yield further valuable insights.

### Conclusions

This study represents one of the earliest investigations into disfluency production patterns in bilingual autistic children. Clinically, our study underscores the pragmatic role of filled pauses (e.g., *eh* and *em*) in bilingual autistic children. Regarding the differentiation between listener- and speaker-oriented disfluencies, our findings suggest that filled pauses tend to be listener-oriented. This assertion is backed by the observation of fewer filled pauses in autistic bilingual children compared to their non-autistic peers and by the link between filled pauses and ToM abilities and working memory.

However, the noted contrast in filled pauses between groups was solely evident in the HL-Russian context. This could be attributed to the fact that the practice of storytelling through picture-based books is more common in kindergartens and schools where societal languages are used, as opposed to at home where the minority language is spoken. This could make storytelling tasks in HL-Russian more challenging and lead to more disfluencies.

The findings of this study emphasize the significance of examining both languages in bilingual children to distinguish between language-specific and language-universal production patterns across languages and clinical groups. It is crucial to consider differences stemming from the varied input and output of each language among bilinguals across diverse activities and contexts in everyday life.

This study contributes to a better understanding of pragmatic abilities in bilingual autistic and non-autistic children and expands our knowledge about potential clinical tools that can help improve conversational skills in this population.

## Supplementary Information

Below is the link to the electronic supplementary material.

Supplementary file 1 (DOCX 74 kb)

## Data Availability

The materials, data, and analysis script for this study can be retrieved from: https://osf.io/256t4/?view_only=9b9948fe0a2c45a4b33da497ee90d237.
