# Supplementary material for: Disfluencies as a Window into Pragmatic Skills in Russian-Hebrew Bilingual Autistic and Non-Autistic Children
Source: J Autism Dev Disord. 2024 Sep 19;56(1):345–61. doi: 10.1007/s10803-024-06533-w (PMC12860835; doi:10.1007/s10803-024-06533-w)
Supplement: Supplementary file 1 — (DOCX 74 kb) [file 10803_2024_6533_MOESM1_ESM.docx]

**Supplementary Information for the paper**

Disfluencies as a Window into Pragmatic Skills in Russian-Hebrew Bilingual Autistic and Non-Autistic Children

Journal of Autism and Developmental Disorders

Authors and Affiliations

[REMOVED for REVIEW]

**Corresponding author**

[REMOVED for REVIEW]

**Online Resource 1**

Table A Comparative Analysis of Disfluency Production in Autistic Children: Key Findings from Various Studies

| Authors (date) | Sample size | Age | Lang. | Disfluency Types | | | | | |
| --- | --- | --- | --- | --- | --- | --- | --- | --- | --- |
|  |  |  |  | FP | SP | Repet. | Correct. | Prol. | Task |
| **Narrative** | | | | | | | | | |
| Irvin et al. (2016) | ASD = 24  TD = 16 | 9-15 | E | ASD < TD  um/fluent words*100 | - | - | - | - | Paint Description |
| Thurber and Tager-Flusberg (1993) | ASD =10  TD =10  10 mildly | 6-13 | E | - | ASD < TDa SP/total words*100 | n.s | n.s | - | Creating a story  ‘Frog, Where Are You?’ by Mercer Mayer (1969). |
| Suh et al. (2014) | ASD = 15  TD = 15 | 9-15 | E | n.s | - | ASD > TD  Repet./utterance | ASD > TD  Correct./utterance | - | Creating a story ‘Tuesday’ (Wiesner, 1991) |
| Kuijper et al. (2016) | ASD = 38  TD = 36 | 6-12 | D | n.s  FP+SP % per syntactic units | | ASD >TD  Repet. % per syntactic units | n.s | - | Creating a story  ‘Tuesday’ (Wiesner, 1991) |
| Palominos-Flores (2019)  (MA thesis) | ASD = 18  TD = 19 | 6-12 | S | ASD < TD | ASD>TDb | - | - | ASD >TD | Creating a story  ‘Tuesday’ (Wiesner, 1991) |
| Grego et al. (2023) | ASD = 42  TD = 26 | 7-11 | E | - | - | n.s | n.s | - | Creating a story  ‘Tuesday’ (Wiesner, 1991) |
| McElroy et al. (2018)  (MA poster ) | ASD = 20  TD = 20 | 11-16 | E | n.s | - | ASD >TD | - | ASD >TD | Creating a story  (Accomplishing) |
| De Marchena and Eigsti (2016) | ASD =18  TD =18 | 12-17 | E | ASD > TD  FP+Repet+Correct. per word  in both conditions | | | | - | Retelling (cartoons)  a. shared condition  b. unshared condition |
| Morett et al. (2016) | ASD = 18  TD = 21 | 10-19 | E | ASD < TD  (in both conditions) | ASD > TDc  (in both conditions) | - | - | - | Retelling (cartoons)  Conditions:  a.visible listener  b. non-visible listener |
| Vidović Zorić and Blažeković, (2023) | ASD =10  TD=10 | 8-16 | C | n.s | ASD > TDd  SP/words*100 | n.s | n.s | n.s | Retelling (cartoons) |
| **Conversation** | | | | | | | | | |
| McGregor and Hadden (2020) | ASD = 31  TD = 32 | 7-15 | E | ASD < TD  *um*/(*um*+*uh*)  *um*/fluent words*100 | - | - | - | - | Interview (favorite game or sport) |
| Jones et al. (2022) | ASD = 21  TD = 20 | 10-17 | E | ASD < TD  *um* ratio | - | - | - | - | Interview (interests and hobbies) |
| Parris-Morish et al. (2016) | ASD = 65  TD = 17 | 6-17 | E | ASD < TD  *um*/(*um*+*uh*) | - | - | - | - | Conversation  (ADOS) |
| Salem et al. (2021) | ASD = 96  TD = 28 | 7-17 | E | ASD < TD  *um* proportion | - | - | - | - | Conversation  (ADOS) |
| Lawley et al. (2022) | ASD =117  TD = 65 | 4-15 | E | ASD < TD  *um* rate  *um*/ratio | - | - | - | - | Conversation  (ADOS) |
| Wiklund and Laakso (2020) | ASD = 5  TD = 6 | 11-13 | F | ASD > TD  disfluencies and ungrammatical expressions/  total duration of speech | | | | | Talk-in-interaction |
| Hallin et al. (2016) | ASD = 7  TD =17 | 7-9 | E | ASD < TD  *eh*+*em*+filler words  /P-unit | - | Repet. +Correct.  n.s | | - | Structured conversation (family, interests, and events) |
| Wong (2019)  (MA thesis) | ASD = 40  TD = 44 | 8-13 | E | ASD > TD  *um* ratio in unstructured task | - | - | - | - | Conversation  a. ‘Expository discourse’ condition  b. ‘Structured conversation’ condition |
| **Activities** | | | | | | | | | |
| Heeman et al. (2010) | ASD = 26  TD = 22 | 4-8 | E | ASD < TD  *um* rate  in all activities | ASD > TDe | - | - | - | ADOS activities: Conversation, Describing a wordless picture or book, Playing with toys |
| Lunsford et al. (2010) | ASD = 22  TD = 26 | 4-8 | E | ASD < TD  *um /* words ratio | - | - | - | - | ADOS activities  Conversation, Describing a wordless picture or book, Playing with toys |
| Gorman et al. (2016) | ASD = 50  TD = 43 | 4 -8 | E | ASD < TD  *um/(um+uh*) ratio  *um*/fluent words | - | - | - | - | ADOS activities  Play, Description of a Picture, Telling a story from a wordless picture book , and Conversation |
| MacFarlane et al. (2017) | ASD = 47  TD =18 | 4 -8 | E | ASD > TD  content disfl. /  (content disfl. +filler words + FP) | - | n.s | n.s | - | ADOS activities:  Play,Description of a Picture, Telling a story from a wordless picture book , and Conversation |
| Tanaka et al. (2014) | ASD = 4  TD = 2 | 10-13 | J | ASD > TD  FP % per total words | ASD > TDe | - | - | - | Doh (free play), jenga (a game), narrative, and natural conversation (activities with parents) |

The data is organized into three main categories: Narrative, Conversation, and Activities, with respective subcategories indicated in the "Task" column. Within each subcategory, studies are arranged chronologically, from earlier to later studies. Age is represented either as an age range in years or as the mean age. A dash (-) indicates that a particular type of disfluency was not observed in the study, and 'n.s' indicates not significant.

Legend: ASD: autistic group, TD: non-autistic group, FP: filled pauses, SP: silent pauses, Repet: Repetitions, Correct: self-corrections, Prol: Prolongations, Lang: Languages (E=English, D=Dutch, J=Japanese, F=Finnish, S=Spanish, C=Croatian).

Note: Participant groups such as SLI (Specific Language Impairment), ADHD (Attention-Deficit/Hyperactivity Disorder), and OO (Optimal Outcome) are not included in this table for brevity.

a difference in non-grammatical (within-phrase) silent pauses longer than 250 milliseconds.

b difference in between-utterance silent pauses longer than 500 milliseconds.

c difference in silent pauses longer than 2 seconds.

d difference in within-utterance/within-phrase silent pauses longer than 120 milliseconds.

e t difference in the length of between-turn silent pauses following a question.

**Online Resource 2.** Results on Cognitive and Mentalizing Standardized Measures for BI-ASD and BI-TD groups in SL-Hebrew

|  | BI-ASD  M (SD) [range]  (n= 21) | BI-TD  M (SD) [range]  (n= 30) | Between-Group differences |
| --- | --- | --- | --- |
| Non-verbal IQ | 24 (6.2) [13-36] | 24 (5.9) [14-36] | t (46) = -0.01, *p* = 1.00 |
| vSTM (FWD) | 3.7 (1.5) [2-8] | 4.2 (1.5) [3-9] | t (45) = -1.16, *p* = .25 |
| vWM (BWD) | 2.5 (1.9) [0-6] | 3.1 (1.1) [2-6] | t (28.08)= -1.12, *p*= .27 |
| ToM Battery | 1.3 (1.1) [0-3] | 2 (0.9) [0-3] | t (46)= - 2.4, ***p***= .02 |

Group comparisons were conducted using independent t-tests. Data are expressed as means and standard deviations. *BI-TD*: bilingual non-autistic children; *BI-ASD:* bilingual autistic children; *FWD:* The Hebrew Forward Digit Span and *BWD*: The Hebrew Backward Digit Span adapted from the Wechsler Intelligence Scale for Children (Wechsler, 1991) were used to assess verbal short-term memory (vSTM) and verbal working memory (vWM); Nonverbal Intelligence Quotient (NVIQ) measured by Raven's colored progression matrices (Raven, 1998); *ToM*: Theory of Mind.

Note 1: Group comparisons were conducted using independent t-tests.

**Online Resource 3.** Results on Standardized Language Measures for BI-ASD and BI-TD Groups in HL-Russian and SL-Hebrew

|  | Task | BI-ASD  Mean(SD) | BI-TD  Mean(SD) | Between-Group differences |
| --- | --- | --- | --- | --- |
| SL-Hebrew | LITMUS Srep-30  (0-1) | 0.61 (0.25) | 0.85 (0.15) | *t* (28.614) = -3.85**, *p* =.0006** |
|  | CLT-Noun Receptive  (0-32) | 29.90 (2.96) | 30.77 (2.42) | *t (*49) = -1.14, *p* = .259 |
|  | CLT-Noun Production  (0-32) | 23.00 (4.07) | 24.37(5.86) | *t* (47) = -0.89, *p* = .379 |
|  | CLT-Verb Receptive  (0-32) | 23.76 (4.39) | 26.87 (3.88) | *t* (49) = -2.66, ***p* = .010** |
|  | CLT-Verb Production  (0-32) | 14.11 (4.93) | 18.03 (5.22) | *t* (47) = -2.62, ***p* = .012** |
| HL-Russian | LITMUS Srep-30  (0-1) | 0.74 (0.27) | 0.87 (0.11) | *t* (23.158) = -2.11, ***p* = .045** |
|  | CLT-Noun Receptive  (0-32) | 28.81(4.82) | 30.00 (1.82) | *t* (49) = -1.2385, *p* = .2214 |
|  | CLT-Noun Production  (0-32) | 21.75 (5.29) | 20.73 (6.88) | *t* (48) = 0.55924, *p* = .5786 |
|  | CLT-Verb Receptive  (0-32) | 25.14 (5.31) | 26.43 (3.43) | *t* (49) = -1.0548, *p* = .2967 |
|  | CLT-Verb Production  (0-32) | 15.30 (5.58) | 14.83 (5.98) | t *t* (48) = 0.27755, *p* = .7825 |

Group comparisons were conducted using independent t-tests. Data are expressed as means and standard deviations. Legend: *BI-TD*: bilingual non-autistic children; *BI-ASD:* bilingual autistic children; *LITMUS SRep-30*: LITMUS Hebrew Sentence Repetition (Armon-Lotem & Meir, 2016; Meir et al., 2016) and LITMUS Russian Sentence Repetition *(Armon-Lotem & Meir, 2016; Meir et al., 2016); CLT-Verb/Noun, Receptive/Production:* subtasks from LITMUS Hebrew Cross-linguistic Task (Altman et al., 2017) and LITMUS Russian Cross-linguistic Task (Gagarina & Nenonen, 2017).

Note 1: **Bold font** indicates a significant difference (*p* < .05).

**Online Resource 4.** Codes for Disfluency Types and Errors based on the Codes for the Human Analysis of Transcripts (CHAT) format

| **Disfluency Type** | **Code** | **Description** | **Examples in HL-Russian and SL-Hebrew** | |
| --- | --- | --- | --- | --- |
| **Silent pauses** | **(.)** | Unfilled pauses | | CHI: *patom ana (.) paShla (Russian)*  %glo: then she (.) went  %tra: then she went |
| **Filled pauses** | **&-** | Non-linguistic sound | | CHI: *hu &-em Raa et ha- Xatul (Hebrew)*  %glo: he &-*um* saw the cat  %tra: he saw the cat |
| **Polysyllabic whole-word repetitions** | **[/]** | Repetition of word with more than one syllable | | CHI: *patom ana [/] ana paShla (Russian)*  %glo: then she (.) went  %tra: then she went |
| **Phrase repetitions** | **< > [/]** | Repetition of more than one words | | CHI: *<a ptitSka moZet> [/] a ptitShka moZet (Russian)*  %glo: <but the bird can> [/] but the bird can  %tra: but the bird can |
| **Whole -word self-corrections** | **[//]** | Self-correction of word | | CHI: *a patom ana pragnala ptitSku &-em [//] l’isu (Russian)*  %glo: then she drove away the bird &-em [//] the fox  %tra: then she drove away the fox |
| **Phrase self-corrections** | **< > [//]** | Self-correction of more than one word | | CHI: *<hajta cipoR &-em> [//] hayu ciporim (Hebrew)*  %glo: there was bird &-em > [//] were birds  %tra: there were birds |
| **Phonological fragments** | **&+** | Abandoned word attempts | | CHI: *ve- pito’m jaca &+ai &+I &+ja (Hebrew)*  %glo: and suddenly came out &+ai &+I &+ya  %tra: and suddenly (the cat) came out &+ai &+I &+ya |
| **Part-word repetitions** | **&+** | Repetition of part of word | | CHI: *pit’om hu &+ha halaX xxx (Hebrew)*  %glo: suddenly he &+ha left xxx  %tra: suddenly he left |
| **Monosyllabic whole-word repetitions** | **[/]** | Repetition of single-syllable word | | CHI: *I [/] i varona (Russian)*  %glo: and [/] and the raven  %tra: and the raven |
| **Prolongations** | **:** | Stretching out vowels within word | | CHI: *a l’isa: b’egala (Russian)*  %glo: and the fo:x was running  %tra: and the fo:x was running |
| **Broken words** | **^** | Pause within word | | CHI: *i on ub’i^Zal (Russian)*  %glo: and he le^ft  %tra: and he left |
| **Errors** | **[*][:**the correct word**]** | Morpho-syntactic and semantic errors | | CHI*: patom ana uganjala [*] [:pragnala] ptichku (Russian)*  %glo: then she drove away [*][:morph.error] the bird %tra: then she drove away the fox |

Note 1: The examples are presented in three lines: the first line is the original utterance in SL-Hebrew or HL-Russian, the second line offers the word-by-word gloss in English, and the third line provides the English translation.

Note 2: Only intra-utterance (c-unit) silent pauses were analyzed.

Note 3: Only prolongations of vowels were analyzed.

Note 4: Part-word repetitions where only a minimal part was left to complete the whole word were marked as shown in the following example: 'slee(p)' with the missing element in parentheses, allowing the CLAN program to count them as whole words.

**Online Resource 5.**

|  | **C-units** | | | | | | | |
| --- | --- | --- | --- | --- | --- | --- | --- | --- |
| *Predictors* | *Estimates* | *std. Error* | *std. Beta* | *standardized std. Error* | *CI* | *standardized CI* | *Statistic* | *p* |
| (Intercept) | 9.78 | 0.64 | -0.12 | 0.23 | 8.52 – 11.05 | -0.57 – 0.33 | 15.37 | **<0.001** |
| Group [BI-TD] | 0.82 | 0.84 | 0.29 | 0.30 | -0.85 – 2.49 | -0.30 – 0.89 | 0.98 | 0.331 |
| Language [Russian] | -0.00 | 0.66 | -0.00 | 0.24 | -1.32 – 1.31 | -0.47 – 0.47 | -0.01 | 0.995 |
| Group [BI-TD] × Language [Russian] | -0.33 | 0.87 | -0.12 | 0.31 | -2.05 – 1.40 | -0.73 – 0.50 | -0.38 | 0.707 |
| Random Effects | | | | | | | | |
| σ^2^ | 4.26 | | | | | | | |
| τ_00_ _Code_ | 3.93 | | | | | | | |
| ICC | 0.48 | | | | | | | |
| N _Code_ | 50 | | | | | | | |
| Observations | 95 | | | | | | | |
| Marginal R^2^ / Conditional R^2^ | 0.015 / 0.487 | | | | | | | |

|  | **Tokens** | | | | | | | |
| --- | --- | --- | --- | --- | --- | --- | --- | --- |
| *Predictors* | *Estimates* | *std. Error* | *std. Beta* | *standardized std. Error* | *CI* | *standardized CI* | *Statistic* | *p* |
| (Intercept) | 56.93 | 5.44 | 0.07 | 0.22 | 46.13 – 67.74 | -0.36 – 0.50 | 10.47 | **<0.001** |
| Group [BI-TD] | 8.47 | 7.16 | 0.34 | 0.28 | -5.76 – 22.70 | -0.23 – 0.90 | 1.18 | 0.240 |
| Language [Russian] | -7.80 | 4.39 | -0.31 | 0.17 | -16.53 – 0.93 | -0.66 – 0.04 | -1.78 | 0.079 |
| Group [BI-TD] × Language [Russian] | -7.59 | 5.78 | -0.30 | 0.23 | -19.06 – 3.89 | -0.76 – 0.15 | -1.31 | 0.193 |
| Random Effects | | | | | | | | |
| σ^2^ | 186.24 | | | | | | | |
| τ_00_ _Code_ | 419.36 | | | | | | | |
| ICC | 0.69 | | | | | | | |
| N _Code_ | 50 | | | | | | | |
| Observations | 95 | | | | | | | |

|  | **Types** | | | | | | | |
| --- | --- | --- | --- | --- | --- | --- | --- | --- |
| *Predictors* | *Estimates* | *std. Error* | *std. Beta* | *standardized std. Error* | *CI* | *standardized CI* | *Statistic* | *p* |
| (Intercept) | 32.09 | 2.47 | 0.12 | 0.22 | 27.19 – 36.99 | -0.33 – 0.56 | 13.00 | **<0.001** |
| Group [BI-TD] | 0.12 | 3.25 | 0.01 | 0.29 | -6.34 – 6.58 | -0.57 – 0.60 | 0.04 | 0.970 |
| Language [Russian] | -2.30 | 2.31 | -0.21 | 0.21 | -6.90 – 2.29 | -0.63 – 0.21 | -1.00 | 0.322 |
| Group [BI-TD] × Language [Russian] | -0.28 | 3.04 | -0.03 | 0.28 | -6.32 – 5.77 | -0.57 – 0.52 | -0.09 | 0.927 |
| Random Effects | | | | | | | | |
| σ^2^ | 51.95 | | | | | | | |
| τ_00_ _Code_ | 71.82 | | | | | | | |
| ICC | 0.58 | | | | | | | |
| N _Code_ | 50 | | | | | | | |
| Observations | 95 | | | | | | | |
| Marginal R^2^ / Conditional R^2^ | 0.012 / 0.585 | | | | | | | |

|  | **Errors** | | | | | | | |
| --- | --- | --- | --- | --- | --- | --- | --- | --- |
| *Predictors* | *Estimates* | *std. Error* | *std. Beta* | *standardized std. Error* | *CI* | *standardized CI* | *Statistic* | *p* |
| (Intercept) | 0.16 | 0.03 | 0.05 | 0.20 | 0.11 – 0.22 | -0.36 – 0.45 | 5.72 | **<0.001** |
| Group [BI-TD] | -0.09 | 0.04 | -0.61 | 0.27 | -0.16 – -0.01 | -1.14 – -0.07 | -2.26 | **0.026** |
| Language [Russian] | 0.08 | 0.03 | 0.59 | 0.23 | 0.02 – 0.15 | 0.14 – 1.04 | 2.61 | **0.011** |
| Group [BI-TD] × Language [Russian] | 0.00 | 0.04 | 0.01 | 0.30 | -0.08 – 0.08 | -0.58 – 0.60 | 0.04 | 0.970 |
| Random Effects | | | | | | | | |
| σ^2^ | 0.01 | | | | | | | |
| τ_00_ _Code_ | 0.01 | | | | | | | |
| ICC | 0.40 | | | | | | | |
| N _Code_ | 50 | | | | | | | |
| Observations | 95 | | | | | | | |
| Marginal R^2^ / Conditional R^2^ |  |  |  |  |  |  |  |  |

**Online Resource 6.**

| **Correlational Analysis for Disfluencies and Language and Cognitive Measures for the BI-TD group in HL-Russian** | | | | | | | | | | | | | | |
| --- | --- | --- | --- | --- | --- | --- | --- | --- | --- | --- | --- | --- | --- | --- |
|  | *SilentPauses* | *FilledPauses* | *PolyWWRepet* | *Prolongations* | *Age* | *SES* | *AOB_HEB* | *Raven* | *FWD* | *BWD* | *ToM* | *CLT_VC* | *SRep* |  |
| *SilentPauses* |  |  |  |  |  |  |  |  |  |  |  |  |  |  |
| *FilledPauses* | -0.23 |  |  |  |  |  |  |  |  |  |  |  |  |  |
| *PolyWWRepet* | 0.02 | -0.11 |  |  |  |  |  |  |  |  |  |  |  |  |
| *Prolongations* | 0.00 | 0.62^***^ | 0.00 |  |  |  |  |  |  |  |  |  |  |  |
| *Age* | 0.13 | 0.02 | -0.22 | -0.09 |  |  |  |  |  |  |  |  |  |  |
| *SES* | -0.07 | 0.04 | -0.00 | -0.08 | -0.30 |  |  |  |  |  |  |  |  |  |
| *AOB_HEB* | 0.02 | -0.23 | -0.23 | -0.27 | 0.21 | -0.00 |  |  |  |  |  |  |  |  |
| *Raven* | 0.11 | 0.08 | -0.16 | -0.10 | 0.72^***^ | -0.16 | 0.27 |  |  |  |  |  |  |  |
| *FWD* | 0.18 | -0.16 | -0.06 | -0.19 | 0.39^*^ | -0.07 | 0.08 | 0.34 |  |  |  |  |  |  |
| *BWD* | 0.41^*^ | -0.02 | 0.04 | -0.16 | 0.43^*^ | -0.07 | 0.06 | 0.58^***^ | 0.56^**^ |  |  |  |  |  |
| *ToM* | 0.22 | 0.03 | -0.43^*^ | -0.10 | 0.36^*^ | -0.34 | 0.17 | 0.27 | 0.31 | 0.20 |  |  |  |  |
| *CLT_VC* | 0.11 | -0.35 | -0.16 | -0.39^*^ | 0.34 | -0.06 | 0.31 | 0.30 | 0.17 | 0.07 | 0.39^*^ |  |  |  |
| *SRep* | -0.19 | -0.06 | -0.14 | -0.19 | 0.23 | -0.28 | 0.14 | 0.11 | 0.59^***^ | 0.26 | 0.44^*^ | 0.26 |  |  |
| *Error ratio* | 0.36 | 0.08 | -0.08 | 0.24 | -0.38^*^ | 0.46^*^ | -0.06 | -0.30 | -0.08 | -0.10 | 0.07 | -0.14 | -0.34 |  |
| *Computed correlation used spearman-method with pairwise-deletion.* | | | | | | | | | | | | | | |

**Online Resource 7.**

| **Correlational Analysis for Disfluencies and Language and Cognitive Measures for the BI-TD group in SL-Hebrew** | | | | | | | | | | | | | | |
| --- | --- | --- | --- | --- | --- | --- | --- | --- | --- | --- | --- | --- | --- | --- |
|  | *SilentPauses* | *FilledPauses* | *PolyWWRepet* | *Prolongations* | *Age* | *SES* | *AOB_HEB* | *Raven* | *FWD* | *BWD* | *ToM* | *CLT_VC* | *SRep* |  |
| *SilentPauses* |  |  |  |  |  |  |  |  |  |  |  |  |  |  |
| *FilledPauses* | 0.05 |  |  |  |  |  |  |  |  |  |  |  |  |  |
| *PolyWWRepet* | 0.22 | 0.12 |  |  |  |  |  |  |  |  |  |  |  |  |
| *Prolongations* | 0.04 | 0.06 | -0.20 |  |  |  |  |  |  |  |  |  |  |  |
| *Age* | 0.61^***^ | -0.02 | -0.00 | 0.27 |  |  |  |  |  |  |  |  |  |  |
| *SES* | -0.06 | -0.02 | -0.07 | 0.06 | -0.30 |  |  |  |  |  |  |  |  |  |
| *AOB_HEB* | 0.67^***^ | -0.26 | -0.09 | -0.12 | 0.21 | -0.00 |  |  |  |  |  |  |  |  |
| *Raven* | 0.59^**^ | 0.07 | 0.08 | 0.27 | 0.72^***^ | -0.16 | 0.27 |  |  |  |  |  |  |  |
| *FWD* | 0.37 | 0.03 | 0.22 | 0.23 | 0.39^*^ | -0.07 | 0.08 | 0.34 |  |  |  |  |  |  |
| *BWD* | 0.35 | 0.03 | 0.03 | 0.35 | 0.43^*^ | -0.07 | 0.06 | 0.58^***^ | 0.56^**^ |  |  |  |  |  |
| *ToM* | 0.23 | -0.03 | -0.13 | -0.11 | 0.36^*^ | -0.34 | 0.17 | 0.27 | 0.31 | 0.20 |  |  |  |  |
| *CLT_VC* | 0.31 | -0.13 | 0.22 | 0.17 | 0.75^***^ | -0.21 | -0.15 | 0.44^*^ | 0.51^**^ | 0.50^**^ | 0.30 |  |  |  |
| *SRep* | 0.36 | -0.06 | 0.20 | 0.06 | 0.32 | -0.12 | 0.04 | 0.17 | 0.71^***^ | 0.32 | 0.19 | 0.56^**^ |  |  |
| *Error ratio* | -0.37 | 0.21 | -0.10 | -0.11 | -0.50^**^ | 0.25 | -0.09 | -0.48^*^ | -0.21 | -0.42^*^ | -0.36 | -0.56^**^ | -0.39^*^ |  |
| *Computed correlation used spearman-method with pairwise-deletion.* | | | | | | | | | | | | | | |

**Online Resource 8.**

| **Correlational Analysis for Disfluencies and Language and Cognitive Measures for the BI-ASD group in HL-Russian** | | | | | | | | | | | | | |
| --- | --- | --- | --- | --- | --- | --- | --- | --- | --- | --- | --- | --- | --- |
|  | *SilentPauses* | *FilledPauses* | *Age* | *SES* | *AOB_HEB* | *ADOS* | *Raven* | *FWD* | *BWD* | *ToM* | *CLT_VC* | *SRep* |  |
| *SilentPauses* |  |  |  |  |  |  |  |  |  |  |  |  |  |
| *FilledPauses* | 0.04 |  |  |  |  |  |  |  |  |  |  |  |  |
| *Age* | 0.02 | 0.24 |  |  |  |  |  |  |  |  |  |  |  |
| *SES* | -0.19 | -0.03 | 0.45^*^ |  |  |  |  |  |  |  |  |  |  |
| *AOB_HEB* | -0.12 | -0.12 | 0.34 | 0.18 |  |  |  |  |  |  |  |  |  |
| *ADOS* | -0.35 | 0.24 | -0.07 | -0.22 | -0.11 |  |  |  |  |  |  |  |  |
| *Raven* | -0.28 | 0.26 | 0.57^**^ | 0.17 | 0.37 | -0.05 |  |  |  |  |  |  |  |
| *FWD* | 0.11 | 0.27 | 0.27 | -0.13 | 0.40 | -0.19 | 0.58^**^ |  |  |  |  |  |  |
| *BWD* | 0.19 | 0.32 | 0.39 | -0.03 | 0.07 | 0.04 | 0.50^*^ | 0.69^***^ |  |  |  |  |  |
| *ToM* | 0.12 | 0.29 | 0.40 | 0.20 | 0.08 | -0.26 | 0.61^**^ | 0.61^**^ | 0.54^*^ |  |  |  |  |
| *CLT_VC* | 0.23 | 0.08 | 0.23 | 0.02 | 0.11 | -0.24 | 0.12 | 0.36 | 0.05 | 0.47^*^ |  |  |  |
| *SRep* | 0.02 | 0.18 | 0.25 | 0.07 | 0.39 | -0.11 | 0.27 | 0.52^*^ | 0.00 | 0.37 | 0.62^**^ |  |  |
| *Error ratio* | -0.07 | -0.04 | -0.35 | -0.06 | -0.24 | 0.22 | -0.44 | -0.53^*^ | -0.32 | -0.71^***^ | -0.71^***^ | -0.52^*^ |  |
| *Computed correlation used spearman-method with pairwise-deletion.* | | | | | | | | | | | | | |

| **Online Resource 9.**  **Correlational Analysis for Disfluencies and Language and Cognitive Measures for the BI-ASD group in SL-Hebrew** | | | | | | | | | | | | | |
| --- | --- | --- | --- | --- | --- | --- | --- | --- | --- | --- | --- | --- | --- |
|  | *SilentPauses* | *FilledPauses* | *Age* | *SES* | *AOB_HEB* | *ADOS* | *Raven* | *FWD* | *BWD* | *ToM* | *CLT_VC* | *SRep* |  |
| *SilentPauses* |  |  |  |  |  |  |  |  |  |  |  |  |  |
| *FilledPauses* | 0.39 |  |  |  |  |  |  |  |  |  |  |  |  |
| *Age* | -0.15 | 0.10 |  |  |  |  |  |  |  |  |  |  |  |
| *SES* | -0.25 | -0.06 | 0.45^*^ |  |  |  |  |  |  |  |  |  |  |
| *AOB_HEB* | -0.33 | 0.02 | 0.34 | 0.18 |  |  |  |  |  |  |  |  |  |
| *ADOS* | 0.10 | 0.01 | -0.07 | -0.22 | -0.11 |  |  |  |  |  |  |  |  |
| *Raven* | -0.25 | 0.26 | 0.57^**^ | 0.17 | 0.37 | -0.05 |  |  |  |  |  |  |  |
| *FWD* | -0.11 | 0.50^*^ | 0.27 | -0.13 | 0.40 | -0.19 | 0.58^**^ |  |  |  |  |  |  |
| *BWD* | 0.26 | 0.49^*^ | 0.39 | -0.03 | 0.07 | 0.04 | 0.50^*^ | 0.69^***^ |  |  |  |  |  |
| *ToM* | -0.04 | 0.46^*^ | 0.40 | 0.20 | 0.08 | -0.26 | 0.61^**^ | 0.61^**^ | 0.54^*^ |  |  |  |  |
| *CLT_VC* | 0.03 | 0.32 | 0.35 | 0.11 | 0.03 | -0.36 | 0.48^*^ | 0.48^*^ | 0.47^*^ | 0.84^***^ |  |  |  |
| *SRep* | -0.02 | 0.39 | 0.24 | 0.29 | 0.16 | -0.23 | 0.37 | 0.36 | 0.24 | 0.85^***^ | 0.75^***^ |  |  |
| *Error ratio* | 0.09 | -0.27 | -0.23 | -0.04 | 0.05 | -0.15 | -0.13 | -0.37 | -0.21 | -0.60^**^ | -0.57^**^ | -0.65^**^ |  |
| *Computed correlation used spearman-method with pairwise-deletion.* | | | | | | | | | | | | | |

**Online Resource 10.**

| **Correlational Analysis for Disfluencies, ADOS and Mentalizing Measures for the BI-ASD group in both languages** | | | | | | | | | | | | |
| --- | --- | --- | --- | --- | --- | --- | --- | --- | --- | --- | --- | --- |
|  | *ADOS* | *ToM* | *SilentPauses* | *FilledPauses* | *MonoWWRepet* | *PolyWWRepet* | *PhRepet* | *PWRepet* | *WWCorrect* | *PhCorrect* | *PhonFrag* |  |
| *ADOS* |  |  |  |  |  |  |  |  |  |  |  |  |
| *ToM* | -0.26 |  |  |  |  |  |  |  |  |  |  |  |
| *SilentPauses* | -0.14 | 0.05 |  |  |  |  |  |  |  |  |  |  |
| *FilledPauses* | 0.12 | 0.36^*^ | 0.19 |  |  |  |  |  |  |  |  |  |
| *MonoWWRepet* | 0.03 | 0.16 | -0.09 | 0.31 |  |  |  |  |  |  |  |  |
| *PolyWWRepet* | 0.04 | -0.10 | 0.13 | -0.16 | -0.18 |  |  |  |  |  |  |  |
| *PhRepet* | 0.02 | -0.25 | 0.07 | 0.02 | 0.24 | 0.06 |  |  |  |  |  |  |
| *PWRepet* | -0.12 | 0.10 | 0.10 | 0.10 | 0.37^*^ | 0.09 | 0.42^**^ |  |  |  |  |  |
| *WWCorrect* | 0.18 | -0.09 | 0.31^*^ | 0.29 | 0.11 | -0.04 | 0.15 | 0.21 |  |  |  |  |
| *PhCorrect* | -0.17 | 0.04 | 0.06 | 0.06 | 0.37^*^ | 0.12 | 0.24 | 0.42^**^ | 0.33^*^ |  |  |  |
| *PhonFrag* | -0.18 | 0.21 | 0.35^*^ | 0.03 | -0.09 | -0.02 | 0.20 | 0.12 | 0.14 | 0.14 |  |  |
| *Prolongations* | -0.08 | -0.17 | 0.13 | -0.02 | 0.14 | 0.17 | 0.25 | -0.00 | -0.05 | 0.11 | 0.30 |  |
| *Computed correlation used spearman-method with pairwise-deletion.* | | | | | | | | | | | | |

| **Online Resource 11.**  **Correlational Analysis for Disfluencies and Mentalizing Standardized Measures for the BI-TD group in both Languages** | | | | | | | | | | | |
| --- | --- | --- | --- | --- | --- | --- | --- | --- | --- | --- | --- |
|  | *ToM* | *SilentPauses* | *FilledPauses* | *MonoWWRepet* | *PolyWWRepet* | *PhRepet* | *PWRepet* | *WWCorrect* | *PhCorrect* | *PhonFrag* |  |
| *ToM* |  |  |  |  |  |  |  |  |  |  |  |
| *SilentPauses* | 0.17 |  |  |  |  |  |  |  |  |  |  |
| *FilledPauses* | 0.06 | 0.09 |  |  |  |  |  |  |  |  |  |
| *MonoWWRepet* | -0.13 | -0.04 | 0.21 |  |  |  |  |  |  |  |  |
| *PolyWWRepet* | -0.25 | 0.31^*^ | 0.08 | 0.04 |  |  |  |  |  |  |  |
| *PhRepet* | -0.08 | -0.07 | 0.04 | 0.14 | -0.07 |  |  |  |  |  |  |
| *PWRepet* | -0.01 | 0.01 | 0.37^**^ | 0.34^*^ | 0.18 | 0.22 |  |  |  |  |  |
| *WWCorrect* | -0.02 | 0.00 | 0.18 | 0.01 | 0.15 | -0.09 | 0.34^*^ |  |  |  |  |
| *PhCorrect* | -0.26 | -0.04 | 0.06 | 0.10 | 0.28^*^ | 0.02 | 0.32^*^ | 0.39^**^ |  |  |  |
| *PhonFrag* | -0.23 | -0.09 | 0.09 | 0.22 | 0.18 | 0.28^*^ | 0.07 | 0.09 | 0.25 |  |  |
| *Prolongations* | -0.10 | -0.20 | 0.12 | 0.20 | -0.27^*^ | 0.12 | 0.03 | 0.15 | 0.15 | 0.15 |  |
| *Computed correlation used spearman-method with pairwise-deletion.* | | | | | | | | | | | |
